# Intervention to optimise body mass index in adolescents and address the triple burden of malnutrition—the Ntshembo (Hope) trial in rural and urban South Africa study: a study protocol for a randomised controlled trial

**DOI:** 10.1186/s13063-026-09535-4

**Published:** 2026-02-12

**Authors:** S. A. Norris, L. K. Micklesfield, N. J. Christofides, S. H. Crouch, D. E. Mathatha, C. Desmond, S. J. Sharp, K. K. Ong, S. M. Tollman, K. Kahn

**Affiliations:** 1 SAMRC Developmental Pathways for Health Research Unit, Department of Paediatrics and Child Health, Faculty of Health Sciences, University of the Witwatersrand, Chris Hani Baragwanath Academic Hospital, 26 Chris Hani Road, Soweto, Johannesburg, South Africa; 2 School of Human Development and Health, Faculty of Medicine, University of Southampton, Southampton, UK; 3 School of Public Health, Faculty of Health Sciences, University of the Witwatersrand, Johannesburg, South Africa; 4 SAMRC Rural Public Health and Health Transitions Research Unit, School of Public Health, Faculty of Health Sciences, University of the Witwatersrand, Johannesburg, South Africa; 5 School of Economics and Finance, Faculty of Commerce, Law and Management, University of the Witwatersrand, Johannesburg, South Africa; 6 MRC Epidemiology Unit, Institute of Metabolic Science, University of Cambridge, Cambridge, UK

**Keywords:** South Africa, Rural, Urban, Adolescence, Malnutrition

## Abstract

**Introduction:**

South Africa faces a complex health burden with burgeoning non-communicable diseases against a background of prevalent infection. The triple burden of malnutrition, comprising undernutrition alongside overweight/obesity and micronutrient deficiencies, is widespread and imposes risks for non-communicable diseases along the life course, especially among adolescent girls. We hypothesise that, by optimising nutrition and body mass index (BMI) of at-risk adolescent girls, we can realise a triple return on investment: improved nutritional status, reduced metabolic risk, and moderated pre-conception exposures to offset transgenerational risk for cardiometabolic disease.

**Methods and analysis:**

We will enrol 1248 girls 14–19 years with either underweight or overweight defined using age-sex-appropriate BMI cut-offs living either in rural or urban South Africa. After baseline assessments and randomisation, participants will be reassessed at 18–24 months follow-up. If a participant becomes pregnant, further assessments will be conducted during pregnancy (< 28 weeks) and postnatally. We will include both process and economic evaluations. The primary outcome is change in BMI standard deviation score from baseline to follow-up aligned to the target direction, i.e. increase in BMI for underweight, decrease in BMI for overweight. Community health workers will deliver the intervention with both household and individual components. A conditional cash transfer will be provided to the household with guidance to improve dietary diversity. Health literacy material, a multi-micronutrient supplement, health screening and support management (for example anaemia; blood pressure; HIV; depression), and facilitating behaviour change to optimise nutrition, physical and mental health will be provided to the adolescent girl.

**Trial registration:**

The trial has been registered with the Pan African Clinical Trials Registry; identifier: PACTR202201638897606. Registered on 1st August 2022. https://pactr.samrc.ac.za/TrialDisplay.aspx?TrialID=14656.

**Supplementary Information:**

The online version contains supplementary material available at 10.1186/s13063-026-09535-4.

## Protocol version {2}

April 2024 (version #2). Any protocol amendments will be communicated to investigators, Human Ethics Research Committees, Trial Steering Committee, Data Safety and Monitoring Board, trial participants, and trial registries.

## Introduction

### Background and rationale {9a}

The economic burden of non-communicable diseases (NCDs) is staggering, especially in low and middle-income countries [1]. Expectations are that within this decade in sub-Saharan Africa, the NCD health burden will surpass that of communicable, maternal and neonatal diseases combined [2, 3]. Malnutrition, decreasing physical activity, increasing sedentary behaviour, poor dietary diversity, and intergenerational effects are factors propagating the rise in NCDs. Globally, we have the largest generation of adolescents and young people in human history and this demands more attention and action so that investments in adolescent nutrition and health can reap a triple dividend (better health for them now, better health into the future, and better health for their future children should they choose to conceive) and a 10-fold economic benefit [4, 5]. Furthermore, the WHO-UNICEF-Lancet Commission declared that the evidence is now clear to place children’s and adolescents’ health, education, and development at the centre of the investment effort [6].

Our research in rural Agincourt in South Africa describes populations in nutritional transition with a high prevalence of stunting (32% of girls by age 1 year) but also adolescent overweight/obesity (33% of young women by age 20 years), central adiposity (35% of 18-year-old girls have central adiposity greater than the recommended adult cut-off), and impaired fasting glucose (5% of 7–15-year-olds) [7, 8]. Comparable data from urban Soweto in South Africa indicate a similar profile of malnutrition: 20% stunting by age 1 year, 47% of 18-year-old girls have excessive central adiposity, and 8% impaired fasting glucose [9]. The longitudinal Birth-to-40 cohort data showed the emergence and tracking of elevated blood pressure through adolescence into adulthood and its association with BMI trajectories [10–12]. In addition, we have found that the prevalence of overweight and obesity in females increased from 10% by age 8 to 43% by age 22 years, while the prevalence in males remained fairly constant into young adulthood at 10%; further girls with obesity by age 5 were 42 times more likely be adults with obesity[9, 10]. The combined effects of early life undernutrition and later rapid weight gain or excess adiposity may exacerbate metabolic disease risk, such as type 2 diabetes (T2D) [13]. Our hypothesis is that by optimising change in BMI of adolescent girls at-risk from malnutrition, we can realise a triple return on investment—improved adolescent nutrition, reduced future metabolic risks, and moderated peri-conceptional exposures that contribute to transgenerational risk for metabolic disease, such as T2D.

### Explanation for the choice of comparator {9b}

Through formative data collected as part of a Joint Global Health Trials Development Grant (MR/P021174/1), we confirmed a significant burden of malnutrition (persisting underweight, high percentages of overweight and obesity, and anaemia) and household food insecurity (refer to Table 1). Evidence from our dietary data suggested that underweight and obesity may have some common underlying causes. Both may be the result of poor-quality diets, undernutrition being the outcome of energy-deficient diets, and obesity the result of consuming large amounts of inexpensive, calorie-dense but nutrient-poor food. Both are the result of households having too little financial resource to afford a diverse and healthy diet. These data suggest that a common intervention focusing on improving dietary quality and diversity might be appropriate irrespective of adolescent body weight. The Lancet Double Burden of Malnutrition series comprehensively illustrated the complex interconnections between inter-generational cycles of energy-deficient and -dense diets, micronutrient deficiencies, and the effects of nutrition transition; rural and urban South Africa exemplify such interactions [14].

**Table 1:** Nutritional status formative data from rural Agincourt and urban Soweto, South Africa

Data collected 2018–2019	Under-weight	Normal weight	Over-weight	Obese
**Preconception (rural Agincourt; mean age 15 years *n* = 762)**				
Nutritional status prevalence based on BMI age-appropriate cut-offs	17%	57%	18%	8%
Household food insecurity prevalence	24% of households			
**Preconception (urban Soweto; mean age 20 years; *n* = 1583)**				
Nutritional status prevalence	6%	48%	24%	22%
Anaemia prevalence (mild-severe)	27%	22%	22%	29%
Household food insecurity prevalence	21%	24%	23%	22%
**Pregnancy (urban Soweto; mean age 22 years; *n* = 817)**				
Nutritional status prevalence at booking (< 18 weeks)	4%	30%	34%	32%
Anaemia prevalence (mild-severe)	34%	32%	23%	21%

Further formative research informed four elements of the *Ntshembo* trial protocol.

- Generalisability: Given the high prevalence of malnutrition in Agincourt and Soweto, we included both rural and urban contexts to increase generalisability to SA and other middle-income settings, to ensure greater policy relevance and scale-up.
- Target population: Pilot work revealed a differential risk by sex; for example, the prevalence of overweight/obesity in both rural and urban females is some three times greater than that of males by late adolescence. Also, following discussions with the Department of Health of South Africa to enhance the potential for scale-up, we would focus on at-risk girls (underweight and overweight) rather than a general adolescent population.
- Study design: Pilot trials in both settings highlighted: (a) challenges associated with cluster randomisation—high mobility of adolescents and their families across cluster communities with a risk of contamination; and (b) weak adherence to community intervention components (peer groups) due to structural constraints. Therefore, we opted for individual randomisation with greater individual and family intervention components to ensure feasibility and improved adherence.
- Intervention components: Formative work highlighted: (a) household structural economic barriers that influence dietary risk patterns in both settings. Our response, aligned with SA social development policy, is to facilitate government social grant uptake and to provide a supplemental household cash transfer. We also showed high levels of anaemia, hence the introduction of a multi-micronutrient component; and disrupted sleep patterns consequent on late night social media use to access free/cheap data, hence an additional focus on sleep. Taking account of lessons learned from pilot work in both settings into study design and intervention development enhances our ability to address important questions. We are unaware of similar trials elsewhere with the potential to do so.

## Objectives {10}

Primary Research Question: In at-risk adolescent girls, can a community health worker (CHW) delivered intervention over 18–24 months involving nutrition (multi-micronutrient supplementation with dietary support) and lifestyle behavioural change, together with a household conditional cash transfer achieve directionally appropriate changes in BMI in underweight (increase BMI) and overweight (decrease BMI) adolescent girls?

Secondary Research Questions:

1. In at-risk adolescent girls, can a community health worker (CHW) delivered intervention over 18–24 months involving nutrition (multi-micronutrient supplementation with dietary support) and lifestyle behavioural change, together with a household conditional cash transfer:
2. Improve micronutrient status in adolescent girls with underweight or overweight?
3. Positively influence individual behaviours relating to diet, physical activity, sedentary behaviours, and sleep?
4. Will these changes taken together be sufficient to impact individual adolescent metabolic disease risk?
5. In those adolescent girls who become pregnant: Will reductions in the variance of BMI impact maternal glucose during pregnancy and subsequent birth weight and adiposity of the newborn?
6. Is the intervention package cost-effective?
7. How can the trial contribute to a national and international strategy to tackle a growing adolescent triple burden of malnutrition and reduce transgenerational risk for metabolic disease such as T2D in rapidly transitioning populations?

## Methods: patient and public involvement, and trial design

### Patient and public involvement {11}

**Fig. 1:**
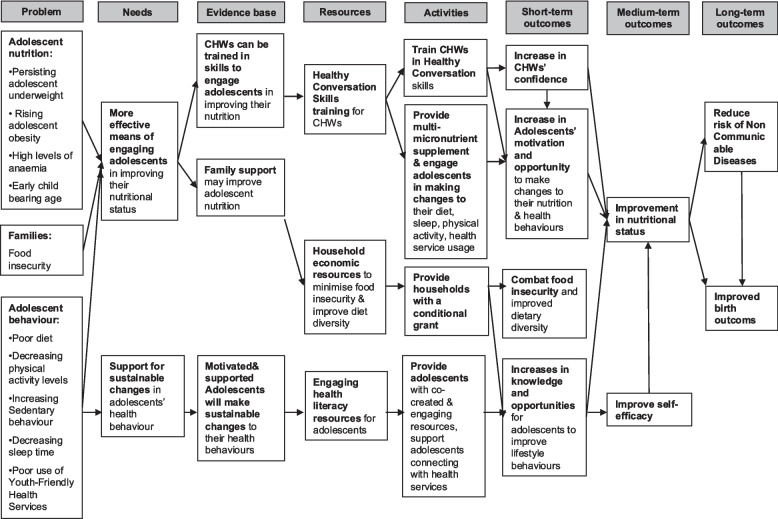
Ntshembo: Logic model for programme of intervention to tackle the double burden of malnutrition in adolescent girls in rural and urban South Africa

We conducted extensive community and adolescent qualitative research exploring a range of topics from nutrition and health, lifestyle, adolescent-friendly health services, and early-stage intervention mapping [15–20]. We also constituted project-specific community advisory groups involving local stakeholders and involved them in the intervention development to ensure its acceptance and feasibility. Specifically, (i) Agincourt village leaders and elected Community Advisory Board and Soweto community forums have agreed to support participation in the trial; (ii) we have demonstrated that community health workers (CHWs) without prior training in behaviour change can acquire the healthy conversation skills (HCS) to support adolescents to improve their nutrition and health behaviours; (iii) we have collected data indicating acceptability and feasibility from adolescents in engaging with the intervention components and materials and on the role of CHWs in supporting them to improve their nutrition and related behaviour. Drawing upon all the formative research, a logic model incorporating theory of change was formulated to underpin the intervention development and delivery (Fig. 1). In 2022, we constituted trial Adolescent Advisory Groups in both Agincourt and Soweto and have involved them in the intervention finalisation and the preparatory phase to enable feedback and input and to facilitate greater acceptance and feasibility of the study. A Stakeholder Advisory Group has been formed with multiple stakeholders (South African government departments of health and education, UNICEF, Save the Children, WHO, NGOs, and academics) to share the development of *Ntshembo* and elicit input and guidance around a variety of issues—policy relevance, linkage with current initiatives, ethics, and dissemination.

### Trial design {12}

It is a primary prevention trial**;** single-blinded individual randomised controlled trial with two arms enrolling adolescent girls aged 14–19 years. Total sample size 1248 girls from both sites, comprising 624 in each arm: treatment and control. Each arm will be stratified by BMI status, underweight and overweight (each 312 girls).

## Methods: participants, interventions, and outcomes

### Trial setting {13}

Two trial-sites within SA will be utilised. The rural location will be the Agincourt area of Bushbuckridge sub-district in northeast SA underpinned by a robust Health and socio-Demographic Surveillance System (HDSS) run by the SAMRC/Wits Rural Public Health and Health Transitions Research Unit. The study area, typical of marginalised former “homelands”, covers 450 km^2^ with some 117,000 people in 22,500 households distributed across 31 adjacent villages (260 people/km^2^) [21]. The Agincourt HDSS has generated over 30 years of longitudinal socio-demographic and vital events data, with experience implementing trials involving adolescents [22, 23]. The urban location of Soweto is an urban-poor area of the City of Johannesburg covering 200 km^2^ with over 1.3 million people (6400 people/km^2^). SAMRC/Wits Developmental Pathways for Health Research Unit has a research centre located at Africa’s largest hospital within Soweto and has 35 years of longitudinal research implementing cohorts and experience of trials within the community [24, 25].

### Characteristics of the people who are needed for the trial

**Table:** 

**Characteristic**	**The people we would expect to see included**
Age	14–19 years
Sex	Female
Gender	Women/Girls
Race, ethnicity and ancestry	African Ancestry including black, coloured and mixed ancestry
Socioeconomic status	Primarily low socioeconomic status
Geographic location	Urban: Soweto, South Africa Rural: Agincourt, South Africa
Other characteristics relevant to the trial	Underweight (defined as BMI < 9th percentile for age by the WHO 2007 international reference) or overweight (BMI 85 to < 95th percentile)

### Eligibility criteria for participants {14a}

#### Inclusion criteria

Adolescent girls who are underweight (defined as BMI < 9th percentile for age by the WHO 2007 international reference) or overweight (BMI 85 to < 95th percentile), aged 14–19 years and a consenting primary caregiver of the index adolescent participant resident in the same household. We envisage that the caregiver is the primary female caretaker, but will also consider the person most responsible for decision-making around the adolescent and purchasing and preparation of food in the household. The adolescent is the primary research participant for the trial outcome, and the caregiver is a secondary participant enrolled for ethical consent, permission, and recipient of the household CCT to enable adherence. The primary and secondary analysis on the adolescent will be conducted independent of the caregiver. Caregiver data will be included as covariates where appropriate.

#### Exclusion criteria

Girls with type 1 diabetes, severe clinical depression, and participants identified with severe intellectual disability will be excluded. Girls living with obesity were excluded given paediatic clinic management.

### Eligibility criteria for sites and those delivering interventions {14b}

The intervention has been informed by the UK MRC Guidelines for Complex Interventions, and we used the TIDieR (Template for Intervention Description and Replication) Checklist. Using the taxonomy of behaviour change techniques (BCTs) [26], we grounded the behaviour change intervention in the theory of planned behaviour, control theory, and social cognitive theory [27–29]. CHWs will be recruited from the study communities and trained to deliver the intervention package. In general, project CHWs will be closely aligned with those aspects of the Department of Health’s Primary Health Care Re-engineering programme (e.g. salary, level of education) that will facilitate future scale-up if Ntshembo proves successful.

### Who will take informed consent? {32a}

Written informed consent will be obtained from all study participants before any data collection or other study procedures commence by a research assistant fluent in the participant’s home language.

### Additional consent provisions for collection and use of participant data and biological specimens {32b}

All data and biological specimens collected will be used solely for the purposes outlined in this protocol and are covered under the primary informed consent process. Should additional sub-studies be conducted, separate informed consent will be obtained from all relevant participants prior to participation.

## Intervention and comparator

### Intervention and comparator description {15a}

#### Intervention components (see Fig. 2)

**Fig. 2:**
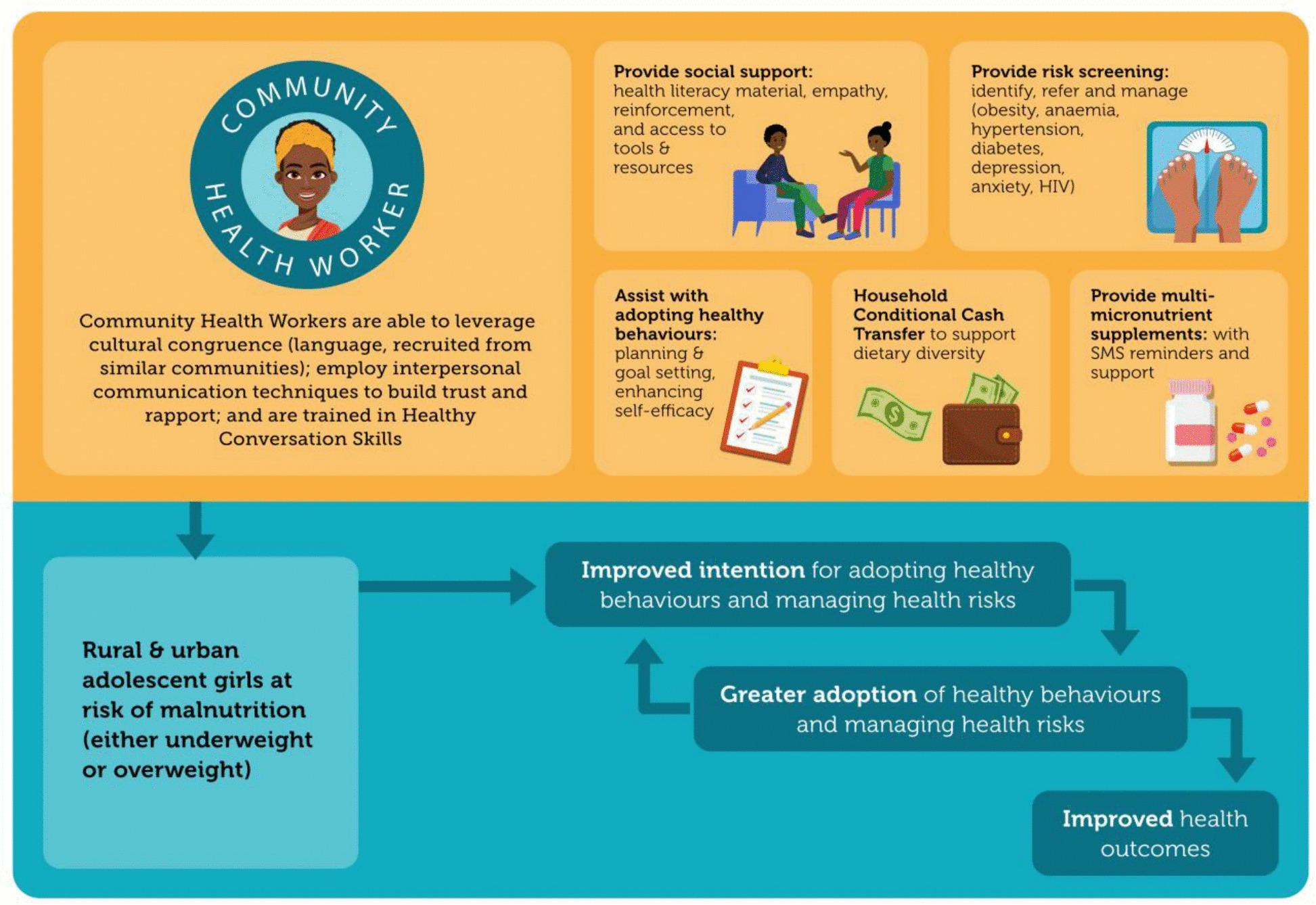
Conceptual outline of the Ntshembo intervention package for underweight or overweight adolescent girls

##### Adolescent-level

1. Health literacy material: In conjunction with topic experts, input and testing with adolescents (specifically involving co-development of materials with the Adolescent Advisory Groups), and a specialised CHW curriculum developer, we integrated behaviour change theory and designed participant materials. The resource book developed for adolescent girls is centred around “living your best life” and contains essential educational content, serial comic-style narrative to convey key concepts, and space for participants to engage with the material through the use of checklists, diaries and reflections. The approach for each module is that of (i) Knowing (what do I know about it?), (ii) Doing (what am I doing about it?), and (iii) Becoming (how do I live my best life?). The resource material encompasses six modules (mental health, stress, making choices about healthy eating, getting strong and healthy (including physical activity and fitness, sitting time), sleep and screens). There is also a module designed for caregivers around dietary diversity.
2. Health Screening: This includes point of care screening, referral and management where appropriate for: anaemia (haemoglobin; Hb), hypertension (elevated blood pressure on three occasions), diabetes (urine dipstick), depression and HIV. Pregnancy testing kits are available on demand.
3. Behaviour change: CHWs will be trained in healthy conversation skills (HCS) [30], a technique developed and tested for use with a range of socio-economically disadvantaged people including youth, to improve self-efficacy and support behaviour change. HCS training aims to achieve basic competencies in skills known to be useful in supporting behaviour change: (i) use of open-discovery questions that lead people to explore and find their own ways to overcome barriers to change; and (ii) the use of SMARTER goal setting (Specific, Meaningful, Achievable, Relevant, Time-bound, Evaluate, Readjust) to provide adolescents with a sense of agency and progress to change lifestyle behaviours. From consultation and recommendation from the Adolesscent Advisory Group, participants will be provided with a personalised print-out from the 7-day activity monitoring, which will be used by the CHW to discuss sleep, physical activity/exercise and sedentary behaviour to help understand their patterns and set appropriate goals.
4. Multi-micronutrient supplement (MMN): The CHW will dispense and monitor the MMN use. The MMN supplement is based on WHO recommendations (Table 2) to improve nutrition, and participants will take the supplement daily. Hb will be monitored regularly (every 6 months) and severely anaemic girls (Hb < 7 g/dL at baseline) will be referred into the public health care system and receive a treatment regime according to current standard of care in SA.

**Table 2:** Nutrient composition of multiple micronutrient supplement

Micronutrient	Unit	Nutrient Reference Value (NRV) EU
Vitamin A	2664 IU	100% (800 µg/2664 IU)
Vitamin D3	200 IU	100% (5 µg/200 IU)
Tocopherol (Vitamin E)	15 mg	125% (12 mg/17.9 I
Vitamin K1	55 µg	73% (75 µg)
Thiamine	1.4 mg	127% (1.1 mg)
Riboflavin	1.4 mg	100% (1.4 mg)
Niacinamide	18 mg	113% (16 mg)
Pantothenic acid	6 mg	100% (6 mg)
Pyridoxine (Vitamin B6)	1.9 mg	136% (1.4 mg)
Biotin	30 µg	60% (50 µg)
Folic acid	600 µg	300% (200 µg)
Cyanocobalamin (Vitamin B12)	2.6 µg	100% (2.5 µg)
Ascorbic acid	60 mg	75% (80mg)
Copper	1.15 mg	100% (1 mg)
Iodine	250 µg	167% (150 µg)
Iron	27 mg	193% (14 mg)
Selenium	50 µg	100% (55 µg)
Zinc	10 mg	100% (10 mg)

##### Family-level

1. Conditional cash-transfer: A monthly cash transfer of ZAR 280.00 (equivalent to ~ £12) will be transferred directly into a bank card issued to the primary caregiver of the adolescent participant co-resident in the same household. The transfer will be monthly, conditional on both caregiver and adolescent engaging with the CHW during their monthly home-based visit. The cash is intended to modestly supplement the child support grant and other government social grants, so enabling poor, food insecure households to make more costly and diverse food choices. “Nudging’ household-level behaviour change may have positive spill-over effects to other members of the household.
2. Health literacy: CHWs will provide caregivers with information and resources around food choices for better nutrition (e.g. the “Eatwell Guide” to improve dietary diversity).
3. Support: CHW will support the primary caregiver to access social grants that they are also eligible to receive.

Adolescents who become pregnant: Those in the treatment arm continue with the intervention. In addition, CHWs will support young women to link early to and regularly attend antenatal services, support parental involvement during pregnancy, and continue the intervention as before the pregnancy. The MMN supplement regimen will continue throughout pregnancy unless the adolescents elect to rather take the antenatal clinic provided supplements. We will provide information for them to share with their antenatal clinic nurses so as to minimise risk of over supplementation.

Control arm with non-health specific intervention: To minimise biases such as special attention given to adolescents in the intervention arm and potentially greater attrition in the control arm, there will be repeated contacts with adolescents in the control arm through a call centre approach (infrastructure in place in both sites). Call centre personnel will deliver a monthly telephonic programme on *Life skills for your future* that will cover the folllowing: (i) Education for your future (importance of education; guidance in selecting school subjects), (ii) Civic rights and responsibilities (how to apply for an identity document; what does it mean to vote?), (iii) Financial education (savings and budgets; how to set up a bank account; applying for an educational bursary), and (iv) Navigating social media (internet; various social media; benefits and harms of social media). Rapid pregnancy testing kits will be freely available for participants. For control participants who become pregnant during the course of the trial, call centre assistants will link them with usual-care antenatal services provided by local primary care clinics.

Duration of treatment period and follow-up: 18–24 months, depending on when baseline data collection is completed. Those who become pregnant in both arms will be followed up to delivery, and their offspring will be measured at or soon after birth.

### Criteria for discontinuing or modifying allocated intervention/comparator {15b}

Any serious unexpected adverse event (SAE) will be reported to Wits University’s Human Research Ethics Committee and Chair of the Data and Safety Monitoring Board; whether or not the adverse event is considered to be related to the trial will be indicated on the submitting form. Where indicated, participants and/or caregivers will be directly linked to care, particularly mental health care services, and not simply referred to care. Participants will be withdrawn if deemed necessary by (i) Wits University’s Human Research Ethics Committee, (ii) the Data and Safety Monitoring Board, (iii) a healthcare provider, (iv) a social worker, or (v) a researcher. No switching between groups may occur; participants will be analysed according to their original randomisation group.

### Strategies to improve adherence to intervention/comparator {15c}

Adherence to the micronutrient supplementation intervention will be monitored and supported at the monthly home visits conducted by a trained CHW. At each visit, the number of MMN dispensed is recorded in a study log. At the subsequent monthly visit, any remaining MMN sachets are collected, counted, and documented prior to dispensing the next month’s supply. Participants are routinely asked whether any supplements were lost or consumed by someone other than themselves. Any such occurrences are recorded. Where non-adherence, loss, or sharing of supplements is reported or identified, the participant receives counselling from the CHW on the importance of adherence to the intervention protocol and the risks associated with sharing supplements.

### Concomitant care permitted or prohibited during the trial {15d}

For participants in the treatment arm, (i) the composition of the multi-micronutrient supplement and potential common side effects (constipation, black stools) will be explained and managed or supplements discontinued if severe; and (ii) for those who become pregnant, over-supplementation is a risk and careful counselling will be given and regularly reinforced explaining that only the study micronutrient supplement should be taken and not the one provided through the antenatal clinic.

### Ancillary and post-trial care {34}

None.

### Outcomes {16}

#### Primary outcome

Change in BMI standard deviation score (SDS) aligned to the target direction—i.e. increase in BMI for underweight, decrease in BMI for overweight—from baseline to follow-up at 18–24 months. As the trial is examining the efficacy of the intervention to address the triple burden of malnutrition (in the underweight group increasing BMI; in the overweight group reducing BMI), the primary outcome variable is both appropriate and innovative.

#### Secondary outcome measures

We will examine the change in anaemia (iron status) as a secondary outcome. Blood pressure and blood glucose will also be key secondary outcomes and will be compared between arms. For those in the intervention group who become pregnant, maternal fasting glucose concentrations around 28 weeks and neonatal adiposity data at delivery (DXA-derived) will be compared with control pregnant participants and their offspring.

### Harms {17}

As most trial participants will be minors, steps will be implemented to protect research participants from any social harms or discomforts and to minimise risks that may be associated with the behavioural interview by ensuring that the study staff make every effort to ensure a comfortable and private environment in which to interact with participants. Should any adolescent < 18 years report abuse, or a CHW suspect abuse of a minor, whether physical or sexual, this will be reported to an appropriate child protection agency or Department of Social Development as legally required. All adverse events will be assessed systematically and reported making use of standard MedDRA language.

### Participant timeline {18}

#### Baseline assessment and randomisation

If eligible, consenting adolescents and their caregivers will enter the trial. Axivity monitors will be given at the home for 7-day monitoring of movement behaviours and undergo baseline assessments (conducted by an independent team of nurses and fieldworkers) at study centres. Thereafter, randomisation and intervention or control activities will begin and last for a minimum of 18 and a maximum of 24 months depending on when recruited.

#### Exit assessment, process, and economic evaluation

Participants will complete the exit assessments. If a girl becomes pregnant during the follow-up period, we will initiate the pregnancy data collection at > 28 weeks of gestation and at delivery. Process and economic evaluation are integral and will be conducted during the course of the trial.

### Sample size {19}

**Fig. 3:**
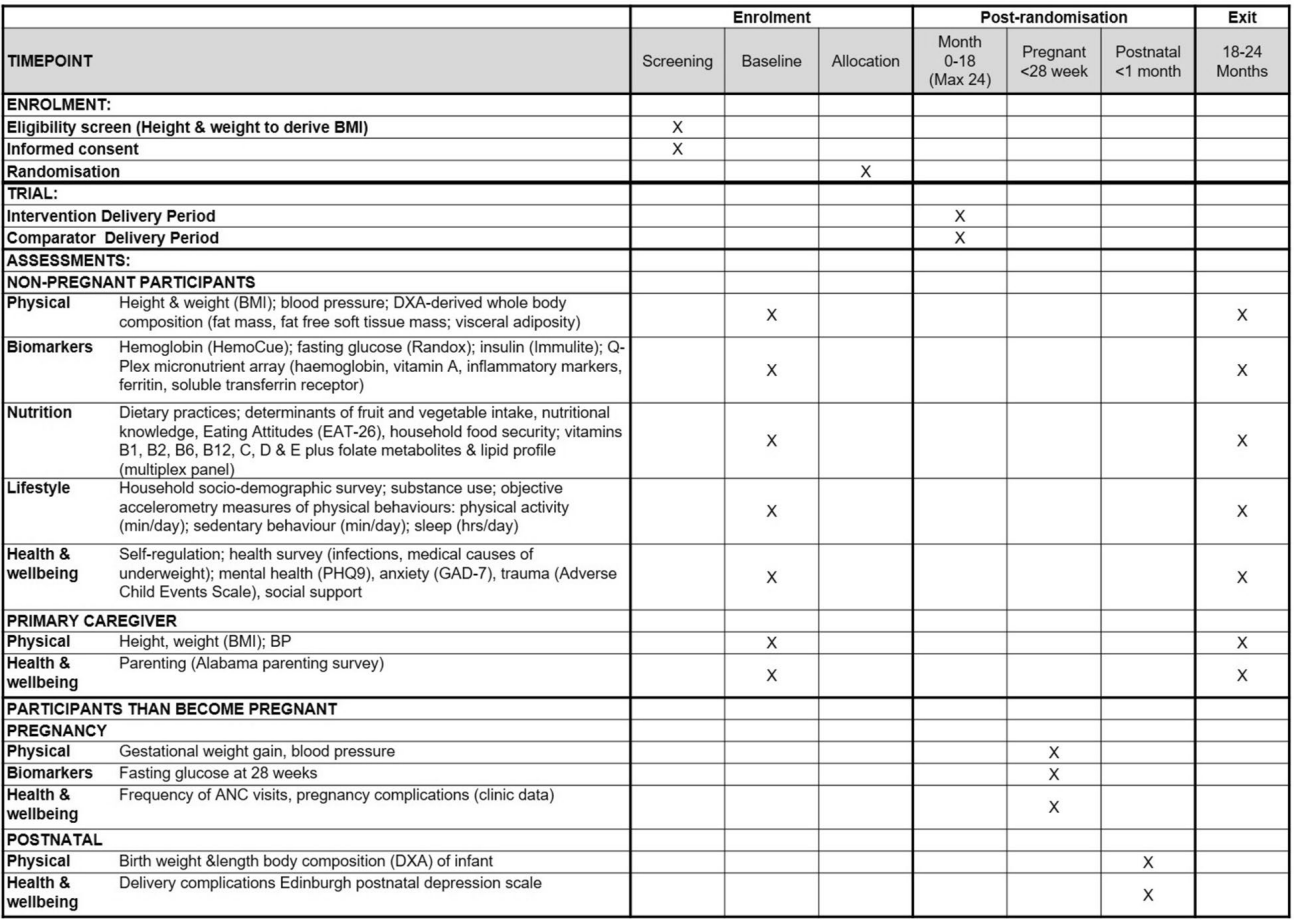
SPIRIT figure study measures, enrolment, interventions, and assessment

For the primary outcome, with a total of 1248 adolescent girls (624 overweight: 312 intervention, 312 control; 624 underweight: 312 intervention, 312 control), there will be 90% power to detect a significant difference in arms of mean target-aligned change in BMI standard deviation score of 0.2 (gain in the underweight group, loss in the overweight/obese group) at the two-sided 5% level (Fig. 3). This assumes a standard deviation of change in BMI SDS of 0.687 calculated using data from adolescent girls in Agincourt assessed at two time points (mean ages 11.5 and 13.6 years), and expected 20% attrition. This degree of change in BMI SDS is equivalent to approximately 2 kg weight loss for girls with overweight and 1 kg higher weight gain in girls with underweight, assuming average height (further details about the sample size calculations, including scenario analyses, in table), compared to controls. For secondary outcomes, the sample also provides 90% power at the two-sided 5% level to detect a 2.1 mmHg difference in systolic blood pressure between intervention and control arms at follow-up, based on a standard deviation of 10 mmHg as reported in young adult black Soweto female adolescents. Regarding the impact of the intervention on pregnancy outcomes, rates of pregnancy in Soweto and Agincourt are high within the age group we plan to study (45.7/1000-person years during the 4-year follow-up period). From the sample of 1248 girls, we therefore anticipate ~ 208 pregnancies, which provides 80% power to detect a difference of 24% of a standard deviation (SD) (Intervention vs. Control) in any parameter at the two-sided 5% level, and 90% power to detect a 27.5% difference. These differences are equivalent to ~ 0.1 mmol/L in maternal fasting glucose at 28 weeks, 0.9–1.0% in neonatal percent body fat, and 0.6–0.7 mm in neonatal sum of skinfolds.

### Recruitment {20}

#### Screening for adolescents at risk

Public engagement teams in Agincourt and Soweto will engage with the Department of Education and high school principals for permission to offer school-based nutrition information sessions followed by anthropometric testing of all 13–19-year-old boys and girls at high school. School-based screening is appropriate as nearly all adolescents attend high school (only ~ 2% not in school), and all schools covering a predefined geographic area will be included to ensure complete ascertainment of potentially eligible participants. Activities will take place on school premises after the school day has ended. Informed consent will be obtained from learners 18 years and older. For learners under 18 years, consent will be obtained from parents/caregivers and assent from the minors themselves. Consent will cover anthropometric measurements at school, as well as the possibility of being contacted at home. The study team will return to participating schools to feedback aggregate results and provide nutrition education to learners and educators.

### Recruitment into trial

Following random sampling of at-risk individuals (identified through school-based screening study) to reach the target number, trained field staff will visit each household inviting them to participate. They will receive verbal and written information in their preferred language and have the opportunity to engage with study staff. We will obtain consent from primary caregivers for their own participation and their minor participant, and from prospective participants aged 18 or 19, as well as assent from participants < 18 years. To avoid coercion on the part of caregivers given the possibility of a cash transfer if randomised to treatment arm, a minor’s refusal to assent will take priority over her caregiver’s consent in discrepant cases.

## Assignment of interventions: randomisation

### Sequence generation: who will generate the sequence {21a}

Randomisation sequence will be generated by the Cambridge Epidemiology and Trials Unit.

### Sequence generation: type of randomisation {21b}

Baseline data collection will be completed on all eligible adolescents who meet the inclusion criteria, after which they will be randomised 1:1 to treatment or control arms. Randomisation will be stratified by location (Agincourt/Soweto) and BMI status (underweight or overweight) and carried out using pre-calculated codes in sealed electronic envelopes displayed on a tablet to ensure allocation concealment.

### Allocation concealment mechanism {22}

The participant herself will “select” between the two sealed options by actively tapping the envelope of choice**.**

### Implementation {23}

The random allocation sequence will be generated by the Cambridge Epidemiology and Trials Unit. Randomisation will be conducted by an independent staff member who is not involved in participant recruitment or intervention delivery. Personnel responsible for enrolling participants and delivering the intervention or comparator will not have access to the random allocation sequence. Following randomisation, participants will be enrolled by the relevant staff assigned to the intervention or control teams according to their allocated group.

## Assignment of interventions: blinding

### Who will be blinded {24a}

Due to the nature of the intervention, trial participants and intervention providers cannot be blinded; however, all researchers, outcome assessors, and data analysts will remain blinded to group allocation.

### How will be blinding be achieved {24b}

Due to the nature of the intervention, participants, intervention providers, and personnel involved in randomisation will be aware of group allocation. Blinding of all other study staff will be maintained through strict separation of trial teams, restricted role-based access to allocation information, controlled data access, and the use of electronic “firewalls” within study databases to prevent inadvertent unblinding.

#### Data collection and management

### Plans for assessment and collection of outcomes {25a}

Measurements will follow strict standard operating protocols that are consistent across both sites. Extensive harmonisation and quality assurance procedures will be in place to ensure similar training of the on-site teams and conduct of data collection. Weight will be measured to the nearest 0.1 kg with a digital scale, and height will be measured to the nearest 1 mm with a stadiometer. Sex and age-specific BMI *z*-scores will be generated for each participant. Waist and hip circumferences will be measured, and ratios computed (waist-to-hip ratio, waist-to-height ratio). Both sites are equipped with Hologic DXA machines, and body composition scans will be done to assess whole body fat, fat-free soft tissue mass, and visceral adiposity. Omron devices will be used to determine systolic and diastolic blood pressure. Autoanalysers or multiplex panels will be used to determine participant biomarker and micronutrient profiles. Dietary intake will be collected using the South African Medical Research Council’s Food-Frequency Questionnaire and food composition tables. Physical activity and sedentary behaviour will be measured using wrist-worn Axivity (AX3; Axivity Ltd, Newcastle Upon Tyne, United Kingdom) monitors worn for 24 h a day for 7 days. Mental health and self-regulation will be assessed by standardised questionnaires appropriate for the study settings. For participants who become pregnant, a pregnancy assessment, including fasting glucose, will be conducted. Infant measures at around 6 weeks postnatal will include neonatal size, circumferences, and skinfold thicknesses according to WHO protocols.

### Economic evaluation

We will conduct a rigorous economic evaluation of the intervention measuring total costs (including provider and beneficiary costs) and use a range of methods to relate costs to benefits. The comparator will be the standard of care observed in the control group. Cost data will be collected from project accounts using a standardised tool and supplemented by additional data on time use, the value of resources used for implementation but not paid for and on participant time costs. Based on these data, we will calculate the total cost of the intervention, estimate the cost of replicating that intervention at a similar scale in similar settings, and model the cost of providing the intervention at scale. The economic evaluation will involve three complementary methods intended to provide a full picture of the relationship between costs and benefits. In the first instance, we will conduct a cost-consequence analysis, where we simply present the costs alongside a vector of benefits. This approach provides a complete summary but makes comparisons on the basis of efficiency and returns on investment difficult. We will, therefore, supplement this analysis with an incremental cost-effectiveness analysis using the primary and secondary outcomes as detailed above, as well as the number of disability-adjusted life years (DALYs) averted by the intervention through the appropriate reduction or increase in BMI. DALYs averted over the life course will be modelled using the change in BMI standard deviation score and information on appropriate confounders. Finally, we will model the long-term implications of the observed benefits, including (depending on the nature and magnitude of benefits observed) later life labour market outcomes. All direct costs for the intervention and control will be calculated as per the study budget, and all indirect costs will be estimated for both intervention and control arms of the trial.

### Process evaluation

Integral to the implementation of complex interventions, we will implement a full process evaluation alongside the trial. A dedicated team will collect data to assess the implementation of the different intervention components over time. Using a mixed-methods approach, we will collect qualitative data from CHWs and the intervention coordinators, and adolescents and their caregivers at three timepoints: baseline, a midpoint, and at the end. Observations of CHW interactions with adolescents and their caregivers will be conducted at multiple time points over the 18–24 months of implementation. Qualitative data will be triangulated with quantitative monitoring data that will document the delivery of all intervention components over the period of implementation. Monitoring data will inform the dose delivered and will include the number and duration of sessions with adolescents and their caregivers. CHW competence and confidence in using Healthy Conversation Skills (HCS) will be assessed by the following: (i) pre- and post-training written assessments and (ii) post-training observations with recorded interviews and reflection on practice. Implementation fidelity, being the extent to which the programme is delivered as planned and the quality of the various components, will assess the delivery of healthy conversations with both pregnant and non-pregnant adolescents and their families. We will observe every CHW monthly during the face-to-face contact sessions with adolescents per site per year, ensuring that all six content sessions are covered (*n* = 55), and sessions with caregivers during the visit (*n* = 55). Observations will also be used to deepen our understanding of how the context might act as a barrier or facilitator to intervention implementation or effects. Detailed field notes of all observations will be written while in the field. In-depth interviews will be conducted with the study coordinators (site manager, treatment arm, and control arm coordinators) at three time points at each site (*n* = 18). Focus group discussions (FGDs) will be held in each site with groups of CHWs at three timepoints over intervention implementation (*n* = 6). The research with project managers and CHWs will focus on their experience implementing the different programme components, including healthy conversation sessions and the supporting resource material, as well as any contextual issues which have enhanced or impeded the implementation of the various intervention components. In order to explore the receptiveness or understanding of the programme by adolescents and their caregivers, as well as possible mechanisms of change, the way in which the intervention components are having an impact on the outcomes or not, will be assessed through qualitative in-depth interviews at multiple time points. We plan to conduct 25 in-depth interviews per site with participants (adolescents and their caregivers) at three time points. All focus groups and interviews will be audio-recorded. We will collect data on the types of goals set and how participants experience progress towards these goals as the findings could inform possible mediators of the study’s primary outcomes.

### Plans to promote participant retention and complete follow-up {25b}

Participant retention will be promoted through monthly contact with participants to maintain engagement, reinforce study importance, and facilitate timely follow-up visits. The use of a generic life skills intervention in the control group is intended to enhance participant engagement and minimise differential attrition between study arms.

### Data management {26}

All data will be gathered electronically, via secure tablets during each visit in both trial sites using the RedCap® data collection and database platform. This system is used successfully in both Soweto and Agincourt-based studies and complies with strict requirements for participant/respondent confidentiality. Data quality/data verification processes will be developed and implemented, including verification of completeness and accuracy of collected data.

### Confidentiality {33}

All participants will be allocated a unique study number for all study information and labelling of results. No personal identification will be displayed in any reports or communication regarding this study. We shall stress and reinforce the critical need for confidentiality by the study teams.

## Statistical methods

### Statistical methods for primary and secondary outcomes {27a}

#### Qualitative data analysis

Audio-recordings from focus groups and interviews will be transcribed verbatim and the transcripts compared with the respective audio-recording to ensure accuracy. Transcripts and field notes will be imported into qualitative data analysis software. Data will be organised into themes, using primarily inductive coding. Using a constant comparative approach, these themes and their sub-themes will be used to produce a coding schema. The relationship between themes and sub-themes will be illustrated in a thematic map. Stages in the analysis will include the following: (a) review of the transcripts; (b) development of a coding scheme to represent emergent themes, supported with verbatim quotations; (c) thematic coding of the transcripts using the coding scheme; (d) development of a thematic map to show a way of viewing the data; and (e) repeating this process (stages a–d) until the thematic map reaches saturation. In order to enhance trustworthiness, 10% of the transcripts will be double-coded to support the development of the coding scheme.

### Statistical analyses

Study results will be reported in accordance with the CONSORT (Consolidated Standards of Reporting Trials) 2010 statement. A statistical analysis plan (SAP) will be prepared and finalised prior to any data analysis. Baseline characteristics will be summarised separately by randomised group. The primary outcome, change in BMI SDS, will be calculated with positive values representing a change in the target direction (i.e. increase in underweight girls, decrease in overweight girls). A linear regression model, adjusted for baseline BMI *z*-score, will be used to estimate the difference in mean change between the intervention and control group, a 95% confidence interval and a *p*-value. The same method will be used to analyse secondary continuous outcomes. We will test for an interaction between randomised group (intervention/control) and BMI group (underweight or overweight/obese), and estimate the intervention effect and 95% confidence interval within each of these groups separately.

### Who will be included in each analysis {27b}

The primary analysis will be a complete-case analysis assuming data are missing completely at random; a sensitivity analysis using multiple imputation (assuming data are missing at random) will be performed; further details about this analysis will be included in the SAP. Complete case analyses will include all participants with full baseline and exit data.

### How missing data will be handled in the analysis {27c}

Levels of missing data will be reported with reasons where available; baseline characteristics will be summarised in participants with and without missing values of the primary outcome.

### Methods for additional analyses (e.g. subgroup analyses) {27d}

We will also analyse the primary outcome within subgroups defined by socio-demographics and levels of compliance with the trial protocol. Each subgroup analysis will be accompanied by the relevant test for interaction. The exact definition of the subgroups will be pre-specified in the trial protocol and SAP.

### Interim analyses {28b}

Interim analyses for the primary outcome would not be informative with regard to early termination of the trial because of the following: (i) we anticipate that recruitment will be completed within a relatively short period; and (ii) the intervention period covers a relatively long (18–24 months) duration to deliver and reinforce the full intervention. Hence, analyses will be conducted at the conclusion of trial data collection. The Data and Safety Monitoring Board will collate and review reported adverse events on a regular basis.

### Protocol and statistical analysis plan {5}

The trial protocol will be made publicly accessible through publication. The statistical analysis plan (SAP) will be available as an attachment to the trial registration and will also be submitted as an update to the published protocol paper prior to database lock.

#### Oversight and monitoring

### Composition of the coordinating centre and trial steering committee {3d}

The coordinating centre comprises the two principal investigators, co-investigators, collaborators, and two trial managers. The Trial Steering Committee (TSC) includes members of the coordinating centre, additional local investigators from each participating site, and a representative from the funding body. The TSC is responsible for decisions regarding trial continuation and protocol amendments, with proposals prepared by the Executive Committee. The TSC Chair receives advice from the independent Data and Safety Monitoring Board (DSMB). The trial managers oversee day-to-day trial operations, are responsible for participant follow-up and overall trial oversight, and report to the members of the coordinating centre weekly.

### Composition of the data monitoring committee, its role and reporting structure {28a}

Safety outcomes will be monitored by an independent Data Safety and Monitoring Board (DSMB). The DSMB comprises a Chief Specialist Scientist at the South African Medical Research Council (SAMRC); a Specialist Statistician and Deputy Unit Director at the Biostatistics Unit, SAMRC; a Paediatrician affiliated with Chris Hani Baragwanath Academic Hospital, Soweto; and a Senior Lecturer in Global Mental Health at the Institute of Psychiatry, Psychology and Neuroscience (IoPPN), King’s College London. The DSMB is independent of the sponsor and funder, and all members serve in an individual capacity with no conflicts of interest.

The primary objective of the DSMB is to safeguard the interests of study participants and to ensure the integrity, scientific validity, and credibility of the Ntshembo trial. Based on cumulative reports and open-session discussions, the DSMB will evaluate participant safety, study conduct, data quality and completeness, and overall trial performance. The DSMB will review the study protocol and any protocol deviations with respect to participant safety and scientific integrity. Recommendations will be communicated to the Chair of the Trial Steering Committee and the Ntshembo Research Committee. Safety monitoring will include assessment of risks inherent to study participation and the impact of any protocol changes on participant risk. Ad-hoc meetings between the DSMB Chair and the Trial Steering Committee may be convened if safety concerns arise (e.g., an unexpected frequency of SAEs). Local trial teams may also refer any SAE of concern directly to the DSMB Chair for consideration.

### Frequency and plans for auditing trial conduct {29}

Trial monitoring will occur at 6-month increments.

### Protocol amendments {31}

Any protocol amendments will be communicated to investigators, Human Ethics Research Committees, Trial Steering Committee, Data Safety and Monitoring Board, trial participants, and trial registries.

### Dissemination policy {8}

The investigator team will publish the results of this trial in academic journals. All data will be presented as group data, rather than individual data. Group results may be disseminated back to participants in the form of a results flyer or community engagement event. Authorship on all publications arising from the trial will follow the international committee of medical journal editors’ criteria. The primary trial outcome paper will be led by the principal investigator, and secondary ancillary analyses will be posed and approved by the study team. The trial will actively promote early career research opportunities for first authorship of papers using trial-generated data.

#### Trial status

Protocol version 2 (April 2024). Between August 2022 and November 2023, we conducted the school survey to aid recruitment into the trial. In collaboration with secondary schools in the Agincourt and Soweto areas, we approached 16,599 adolescent boys and girls and managed to consent and measure height and weight on 12,644 adolescents. From this school survey, we generated a database of underweight and overweight adolescent girls for potential recruitment into the Ntshembo RCT. When then executed a short pilot and found procedures feasible, but we did make some adjustments: (i) added the remeasurement of height and weight at home at the point of enrolment to verify BMI, and if the participant had shifted into either normal or obese weight ranges, they were provided with information and a participation certificate, and thanked them for their time, but not enrolled into the trial; (ii) refined our training and communication processes of the field team around the trial consent with participants to ensure better understanding and capturing information if disclosed on any refusals. Given these minor adjustments, all enrolled participants in the pilot phase were transferred to the main trial which commenced in May 2024. Recruitment of adolescent girls into the trial has been completed, and we anticipate that the last patient/last visit will be in June 2026. We elected to submit later until the protocol was fully stabilised and no unanticipated issue around the adolescents’ safety/wellbeing directly related to the trial was detected. Also, to ensure that the statistical analysis plan was finalised, and that this with trial recruitment progress and reported severe adverse events over a sufficient time period, were all reviewed and signed off by both the Trial Steering Committee and the Data Safety and Monitoring Board (see Additional file 2).

## Supplementary Information

Additional file 1: SPIRIT checklist.

Additional file 2: Statistical Analysis Plan.

## Data Availability

All data will be available within 6 months from the end of the trial and metadata will be open access through request to any of the investigators.

## Abbreviations

ANC: Antinatal care
BCT: Behaviour change techniques
BMI: Body mass index
BP: Blood pressure
CHW: Community healthcare worker
CONSORT: Consolidated Standards of Reporting Trials
DHSC: Department of Health and Social Care
DXA: Dual-energy X-ray absorptiometry
EAT-26: Eating Attitudes Test—26
FCDO: Foreign, Commonwealth & Development Office
FGD: Focus group discussion
GAD-7: Generalised anxiety disorder
Hb: Haemoglobin
HCS: Healthy conversations skills
HDSS: Health and Socio-Demographic Surveillance System
HIV: Humam immunodeficiency virus
MMN: Multi micronutrients
MRC: Medical Research Council
NCD: Non-communicable disease
PHQ-9: Patient Health Questionnaire 9
RCT: Randomised controlled trial
SA: South Africa
SAE: Severe adverse event
SAMRC: South African Medical Research Council
SAP: Statistical analysis plan
SAPRIN: South African Population Research Infrastructure Network
SD: Standard deviation
SDS: Standard deviation score
SMARTER: Specific, Meaningful, Achievable, Relevant, Time-bound, Evaluate, Readjust
SPIRIT: Standard Protocol Items Recommendations for Interventional Trials
T2D: Type 2 diabetes
TIDieR: Template for Intervention Description and Replication
ZAR: South African Rands

## References

[1] García-Morales C, Heredia-Pi I, Guerrero-López CM, et al. Social and economic impacts of non-communicable diseases by gender and its correlates: a literature review. Int J Equity Health. 2024;23:274. 39736607 10.1186/s12939-024-02348-4 PMC11686863

[2] Gouda HN, Charlson F, Sorsdahl K, Ahmadzada S, Ferrari AJ, Erskine H, et al. Burden of non-communicable diseases in sub-Saharan Africa, 1990-2017: results from the Global Burden of Disease Study 2017. Lancet Glob Health. 2019;7(10):e1375–87. 31537368 10.1016/S2214-109X(19)30374-2

[3] Barry A, Impouma B, Wolfe CM, Campos A, Richards NC, Kalu A, et al. Non-communicable diseases in the WHO African region: analysis of risk factors, mortality, and responses based on WHO data. Sci Rep. 2025;15(1):12288. 40210980 10.1038/s41598-025-97180-3 PMC11986008

[4] Patton GC, Sawyer SM, Santelli JS, Ross DA, Afifi R, Allen NB, et al. Our future: a Lancet commission on adolescent health and wellbeing. Lancet. 2016;387(10036):2423–78. 27174304 10.1016/S0140-6736(16)00579-1 PMC5832967

[5] Norris SA, Frongillo EA, Black MM, Dong Y, Fall C, Lampl M, et al. Nutrition in adolescent growth and development. Lancet. 2022;399(10320):172–84. 34856190 10.1016/S0140-6736(21)01590-7

[6] Clark H, Coll-Seck AM, Banerjee A, Peterson S, Dalglish SL, Ameratunga S, et al. A future for the world’s children? A WHO-UNICEF-Lancet Commission Lancet. 2020;395(10224):605–58. 10.1016/S0140-6736(19)32540-1 32085821

[7] Kimani-Murage EW, Kahn K, Pettifor JM, Tollman SM, Dunger DB, Gómez-Olivé XF, et al. The prevalence of stunting, overweight and obesity, and metabolic disease risk in rural South African children. BMC Public Health. 2010;25(10):158. 10.1186/1471-2458-10-158 PMC2853509 20338024

[8] Kimani-Murage EW, Kahn K, Pettifor JM, Tollman SM, Klipstein-Grobusch K, Norris SA. Predictors of adolescent weight status and central obesity in rural South Africa. Public Health Nutr. 2011;14(6):1114–22. 21356151 10.1017/S1368980011000139 PMC3370923

[9] Nyati LH, Pettifor JM, Norris SA. The prevalence of malnutrition and growth percentiles for urban South African children. BMC Public Health. 2019;19(1):492. 31046727 10.1186/s12889-019-6794-1 PMC6498578

[10] Kagura J, Adair LS, Musa MG, Pettifor JM, Norris SA. Blood pressure tracking in urban black South African children: birth to twenty cohort. BMC Pediatr. 2015;15(15):78. 26173634 10.1186/s12887-015-0402-z PMC4502542

[11] Munthali RJ, Kagura J, Lombard Z, Norris SA. Childhood adiposity trajectories are associated with late adolescent blood pressure: birth to twenty cohort. BMC Public Health. 2016;29(16):665. 10.1186/s12889-016-3337-x PMC4966706 27473865

[12] Naidoo S, Kagura J, Fabian J, Norris SA. Early life factors and longitudinal blood pressure trajectories are associated with elevated blood pressure in early adulthood. Hypertension. 2019;73(2):301–9. 30580689 10.1161/HYPERTENSIONAHA.118.11992

[13] Norris SA, Osmond C, Gigante D, Kuzawa CW, Ramakrishnan L, Lee NR, et al. Size at birth, weight gain in infancy and childhood, and adult diabetes risk in five low- or middle-income country birth cohorts. Diabetes Care. 2012;35(1):72–9. 22100968 10.2337/dc11-0456 PMC3241316

[14] Wells JC, Sawaya AL, Wibaek R, Mwangome M, Poullas MS, Yajnik CS, et al. The double burden of malnutrition: aetiological pathways and consequences for health. Lancet. 2020;395(10217):75–88. 31852605 10.1016/S0140-6736(19)32472-9 PMC7613491

[15] Sedibe HM, Kahn K, Edin K, Gitau T, Ivarsson A, Norris SA. Qualitative study exploring healthy eating practices and physical activity among adolescent girls in rural South Africa. BMC Pediatr. 2014;26(14):211. 10.1186/1471-2431-14-211 PMC4150418 25164604

[16] Geary RS, Gómez-Olivé FX, Kahn K, Tollman S, Norris SA. Barriers to and facilitators of the provision of a youth-friendly health services programme in rural South Africa. BMC Health Serv Res. 2014;16(14):259. 10.1186/1472-6963-14-259 PMC4067688 24934095

[17] Draper CE, Micklesfield LK, Kahn K, et al. Application of intervention mapping to develop a community-based health promotion pre-pregnancy intervention for adolescent girls in rural South Africa: project Ntshembo (Hope). BMC Public Health. 2014;14(Suppl 2):S5. 25080940 10.1186/1471-2458-14-S2-S5 PMC4120156

[18] Geary RS, Webb EL, Clarke L, Norris SA. Evaluating youth-friendly health services: young people’s perspectives from a simulated client study in urban South Africa. Glob Health Action. 2015;8:26080. 10.3402/gha.v8.26080. 25623610 10.3402/gha.v8.26080 PMC4306747

[19] Twine R, Kahn K, Scholtz A, Norris SA. Involvement of stakeholders in determining health priorities of adolescents in rural South Africa. Glob Health Action. 2016;9(15):29162. 10.3402/gha.v9.29162. 26983724 10.3402/gha.v9.29162 PMC4794732

[20] Seabi TM, Wagner RG, Norris SA, Tollman SM, Twine R, Dunger DB, et al. Adolescents’ understanding of obesity: a qualitative study from rural South Africa. Glob Health Action. 2021;14(1):1968598. 34482795 10.1080/16549716.2021.1968598 PMC8425773

[21] Kahn K, Collinson MA, Gómez-Olivé FX, Mokoena O, Twine R, Mee P, et al. Profile: Agincourt health and socio-demographic surveillance system. Int J Epidemiol. 2012;41(4):988–1001. 22933647 10.1093/ije/dys115 PMC3429877

[22] Pettifor A, MacPhail C, Hughes JP, Selin A, Wang J, Gómez-Olivé FX, et al. The effect of a conditional cash transfer on HIV incidence in young women in rural South Africa (HPTN 068): a phase 3, randomised controlled trial. Lancet Glob Health. 2016;4(12):e978–88. 27815148 10.1016/S2214-109X(16)30253-4 PMC5626439

[23] Moffet BD, Pozuelo JR, van Heerden A, O’Mahen H, Craske M, Sodi T, et al. Digital delivery of behavioral activation therapy to overcome depression and facilitate social and economic transitions in adolescents in South Africa (the DOBAt study): protocol for a randomised controlled trial. BMJ Open. 2022;12:e065977. 10.1136/bmjopen-2022-065977 PMC9809228 36585150

[24] Richter L, Norris S, Pettifor J, Yach D, Cameron N. Cohort profile: Mandela’s children: the 1990 birth to twenty study in South Africa. Int J Epidemiol. 2007;36(3):504–11. 10.1093/ije/dym016. 17355979 10.1093/ije/dym016 PMC2702039

[25] Norris SA, Draper CE, Prioreschi A, Smuts CM, Ware LJ, Dennis C, et al. Building knowledge, optimising physical and mental health and setting up healthier life trajectories in South African women (*Bukhali*): a preconception randomised control trial part of the Healthy Life Trajectories Initiative (HeLTI). BMJ Open. 2022;12(4):e059914. 35450913 10.1136/bmjopen-2021-059914 PMC9024255

[26] Abraham C, Michie S. A taxonomy of behavior change techniques used in interventions. Health Psychol. 2008;27(3):379–87. 18624603 10.1037/0278-6133.27.3.379

[27] Ajzen I. The theory of planned behavior. Organ Behav Hum Decis Process. 1991;50(2):179–211.

[28] Carver CS, Scheier MF. Control theory: a useful conceptual framework for personality-social, clinical, and health psychology. Psychol Bull. 1982;92(1):111–35. 7134324

[29] Bandura A. Social foundations of thought and action: a social cognitive theory. Englewood Cliffs, NJ: Prentice-Hall; 1986.

[30] Lawrence W, Black C, Tinati T, Cradock S, Begum R, Jarman M, et al. ‘Making every contact count’: evaluation of the impact of an intervention to train health and social care practitioners in skills to support health behaviour change. J Health Psychol. 2016;21(2):138–51. 24713156 10.1177/1359105314523304 PMC4678584

