# Supplementary material for: Intervention to optimise body mass index in adolescents and address the triple burden of malnutrition—the Ntshembo (Hope) trial in rural and urban South Africa study: a study protocol for a randomised controlled trial
Source: Trials. 2026 Feb 12;27:212. doi: 10.1186/s13063-026-09535-4 (PMC12998280; doi:10.1186/s13063-026-09535-4)
Supplement: Supplementary file 2 — Additional file 2: Statistical Analysis Plan. [file 13063_2026_9535_MOESM2_ESM.docx]

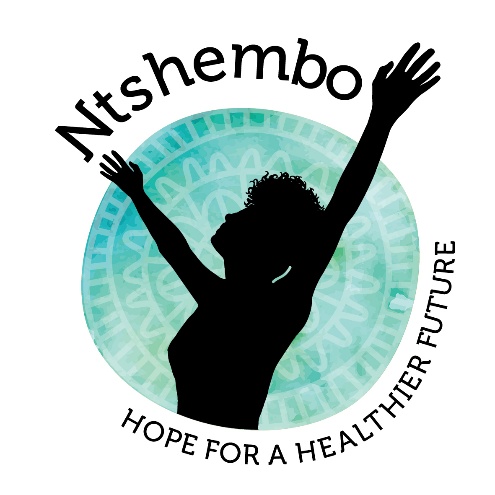
**Statistical Analysis Plan (SAP)**

**Authors:**

Simone H Crouch^1^, Stephen J Sharp^2^, Shane A Norris^1,3^

**Affiliations:**

^1^ SAMRC/Wits Developmental Pathways for Health Research Unit, Department of Paediatrics and Child Health, Faculty of Health Sciences, University of the Witwatersrand, South Africa

^2^ MRC Epidemiology Unit, University of Cambridge, UK

^3^ School of Human Development and Health, Faculty of Medicine, University of Southampton, UK.

Corresponding author: Shane Norris


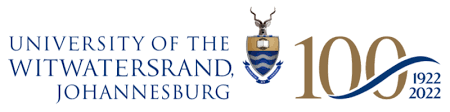

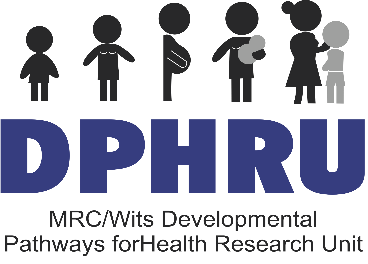


# List of abbreviations

ACE Adverse childhood event

BP Blood Pressure

BMI Body Mass Index

CI Confidence interval

DBP Diastolic Blood Pressure

DXA Dual-energy X-ray absorptiometry

DiD Difference in Differences

FMI Fat Mass Index

HbA1c Glycated haemoglobin

HCS Healthy Conversation Skills

HIV Human Immunodeficiency-virus

IQR Interquartile Range

ITT Intention-to-treat

MAR Missing at Random

MCAR Missing Completely At Random

MI Multiple Imputation

NCD Non-communicable disease

OWO Overweight / Obesity

RCT Randomised Controlled Trial

SAP Statistical Analysis Plan

SD Standard Deviation

SBP Systolic Blood Pressure

# Abstract

The Ntshembo trial is a Phase II randomised controlled trial evaluating the cumulative effect of a complex, multicomponent intervention in adolescent girls in Soweto, South Africa. The trial’s primary endpoint is change in BMI standard deviation score (SDS) aligned to the target direction – i.e. increase in BMI for underweight, decrease in BMI for overweight – from baseline to follow-up at 18-24 months. This Statistical Analysis Plan (SAP) documents the trial background and, specifically, prespecifies the analytic framework for the Ntshembo trial. It defines analysis populations, outcomes, statistical models, handling of missing data, multiplicity, and details planned sensitivity and subgroup analyses.

**Keywords**: Preconception health, multifaceted intervention, low- and middle-income setting, statistical analysis plan

**Contents**

[1 List of abbreviations 2](#_Toc209093064)

[2 Abstract 2](#_Toc209093065)

[3 Administrative information 4](#_Toc209093066)

[3.1 Study identifiers 4](#_Toc209093067)

[3.2 Revision history 4](#_Toc209093068)

[3.3 Contribution and approvals 4](#_Toc209093069)

[4 Introduction 5](#_Toc209093072)

[4.1 Study synopsis 5](#_Toc209093073)

[4.2 Study population 5](#_Toc209093074)

[4.2.1 Inclusion criteria 5](#_Toc209093075)

[4.2.2 Exclusion criteria 5](#_Toc209093076)

[4.3 Interventions 5](#_Toc209093077)

[4.4 Study visits 7](#_Toc209093078)

[4.5 Outcomes 10](#_Toc209093079)

[4.5.1 Primary outcome 10](#_Toc209093080)

[4.5.2 Secondary outcomes](#_Toc209093081) 11

[4.5.3 Safety outcomes 10](#_Toc209093082)

[4.6 Randomisation and blinding 11](#_Toc209093083)

[4.7 Sample size 11](#_Toc209093084)

[5 Statistical analysis 11](#_Toc209093085)

[5.1 Statistical hypotheses 11](#_Toc209093086)

[5.2 Statistical principles 11](#_Toc209093087)

[5.2.1 Level of statistical significance 12](#_Toc209093088)

[5.2.2 Statistical software 12](#_Toc209093089)

[5.3 Analysis plan 12](#_Toc209093090)

[5.5.2 Adjusted analysis 14](#_Toc209093094)

# Administrative information

## Study identifiers

*Ntshembo* has ethical approval from the Human Research Ethics Committee of the University of the Witwatersrand, Johannesburg, South Africa (M211061) and of the University of Cambridge (HBREC.2022.07). This trial is registered with the Pan African Clinical Trials Registry (<https://pactr.samrc.ac.za>); identifier: PACTR202201638897606Revision history

| **Version** | **Date** | **Details** |
| --- | --- | --- |
| 1.0 (draft) | September 2025 | First full draft of Statistical Analysis Plan of the Ntshembo trial |
| 1.1 (revised draft) | October 2025 | Revised draft |
| 1.2 (final version) | November 2025 | Final version signed off by Trial Steering Committee and Data Safety Monitoring Board |

# Introduction

## Synopsis

The Ntshembo trial is an individually randomised controlled trial designed to evaluate the cumulative effect of a complex, multicomponent intervention initiated in adolescents on BMI standard deviation score (SDS) aligned to the target direction South Africa faces a complex health burden with burgeoning non-communicable diseases against a background of prevalent infection. This trial enrolled 1248 girls 14-19y with either underweight or overweight defined using age-sex-appropriate BMI cut-offs living either in rural or urban South Africa. Participants were randomly allocated (1:1) to an intervention or control arm. The intervention, delivered by trained “health helpers” comparable to community health workers. After baseline assessments and randomisation, participants will be reassessed at 18-24 months follow-up. If a participant becomes pregnant, further assessments will be conducted during pregnancy (<28weeks) and postnatally. We will include both process and economic evaluations. The primary outcome is change in BMI standard deviation score from baseline to follow-up aligned to the target direction, i.e. increase in BMI for underweight, decrease in BMI for overweight. Community health workers will deliver the intervention with both household and individual components. A conditional cash transfer will be provided to the household with guidance to improve dietary diversity. Health literacy material, a multi-micronutrient supplement, health screening and support management (for example anaemia; blood pressure; HIV; depression), and facilitating behaviour change to optimise nutrition, physical and mental health will be provided to the adolescent girl. The control group received monthly telephone-based life skills sessions alongside routine health care, HIV, and pregnancy testing.

## Study population

### Inclusion criteria

- Adolescent girls who are underweight (defined as BMI < 9^th^ percentile for age by the WHO 2007 international reference) or overweight (BMI 85 to < 95^th^ percentile)
- Aged 14-19y
- A consenting primary caregiver of the index adolescent participant resident in the same household.

### Exclusion criteria

## Girls with Type-1 diabetes

## Participants with severe clinical depression

## Participants identified with severe intellectual disability

## Girls living with obesity were excluded given paediatric clinic management.

## Interventions

**Intervention components (see Figure 2)**

**Adolescent-level**

1. **Health literacy material:** In conjunction with topic experts, input and testing with adolescents (specifically involving co-development of materials with the Adolescent Advisory Groups), and a specialised CHW curriculum developer, we integrated behaviour change theory and designed participant materials. The resource book developed for adolescent girls is centred around “living your best life” and contains essential educational content, serial comic-style narrative to convey key concepts, and space for participants to engage with the material through the use of checklists, diaries and reflections. The approach for each module is that of: (i) Knowing (what do I know about it?), (ii) Doing (what am I doing about it?), and (iii) Becoming (how do I live my best life?). The resource material encompasses six modules (mental health, stress, making choices about healthy eating, getting strong and healthy (including physical activity and fitness, sitting time), sleep and screens). There is also a module designed for caregivers around dietary diversity.
2. **Health Screening:** This includes point of care screening, referral and management where appropriate for: anaemia (haemoglobin; Hb), hypertension (elevated blood pressure on three occasions), diabetes (urine dipstick), depression, and HIV). Pregnancy testing kits are available on demand.
3. **Behaviour change:** CHWs will be trained in healthy conversation skills (HCS)^30^, a technique developed and tested for use with a range of socio-economically disadvantaged people including youth, to improve self-efficacy and support behaviour change. HCS training aims to achieve basic competencies in skills known to be useful in supporting behaviour change: (i) use of open-discovery questions that lead people to explore and find their own ways to overcome barriers to change; and (ii) the use of SMARTER goal setting (Specific, Meaningful, Achievable, Relevant, Time-bound, Evaluate, Readjust) to provide adolescents with a sense of agency and progress to change lifestyle behaviours. From consultation and recommendation from the Adolesscent Advisory Group, participants will be provided with a personalised print-out from the 7-day activity monitoring, which will be used by the CHW to discuss sleep, physical activity/exercise and sedentary behaviour to help understand their patterns and set appropriate goals.
4. **Multi-micronutrient supplement (MMN):** The CHW will dispense and monitor the MMN use. The MMN supplement is based on WHO recommendations (Table 2) to improve nutrition, and participants will take the supplement daily. Hb will be monitored regularly (every 6-months) and severely anaemic girls (Hb <7g/dL at baseline) will be referred into the public heath care system and receive a treatment regime according to current standard of care in SA.

*
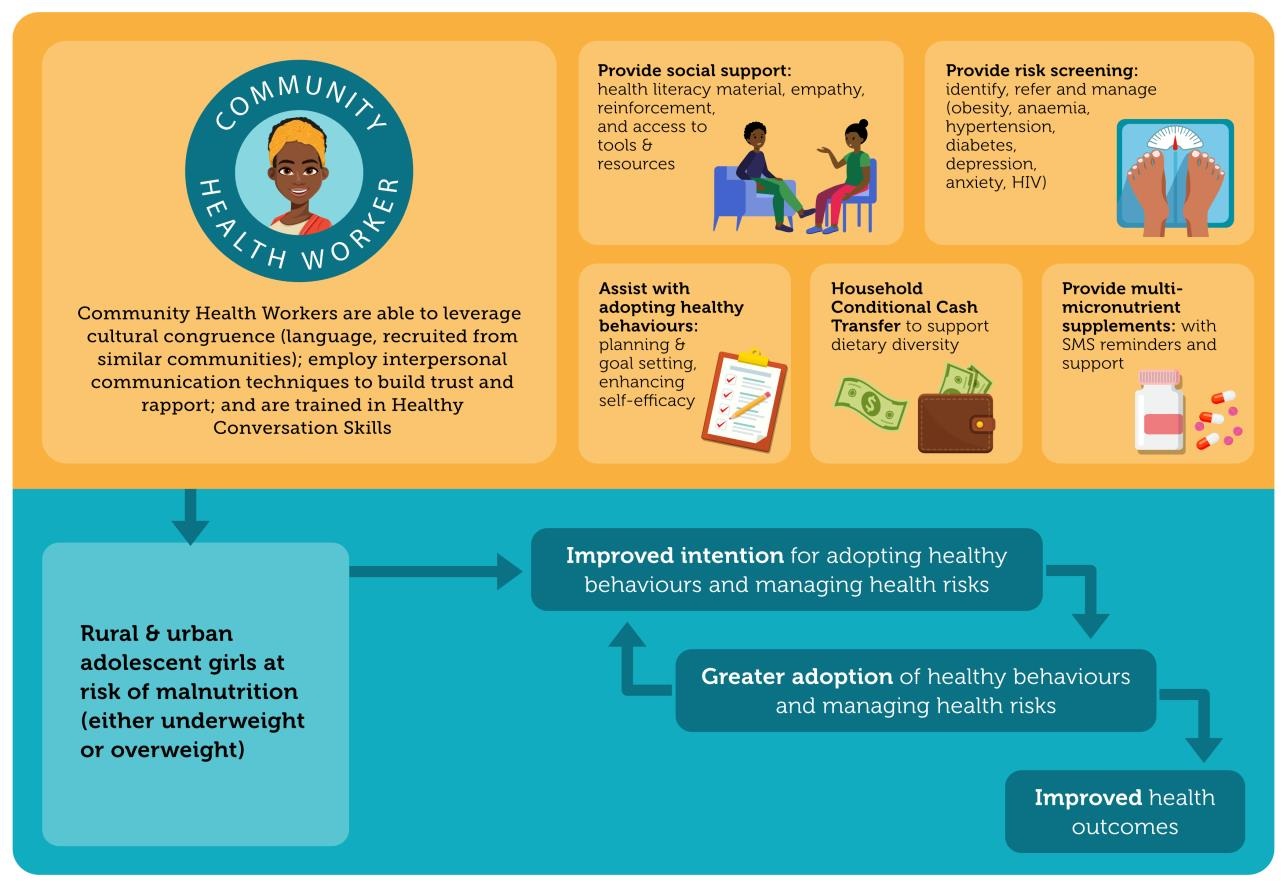
*

| **Table 2: Nutrient composition of multiple micronutrient supplement** | | |
| --- | --- | --- |
| **Micronutrient** | **Unit** | **Nutrient Reference Value (NRV) EU** |
| Vitamin A | 2664 IU | 100% (800 µg / 2664 IU) |
| Vitamin D3 | 200 IU | 100% (5 µg / 200 IU) |
| Tocopherol (Vitamin E) | 15 mg | 125% (12 mg / 17.9 I |
| Vitamin K1 | 55 µg | 73% (75 µg) |
| Thiamine | 1.4 mg | 127% (1.1 mg) |
| Riboflavin | 1.4 mg | 100% (1.4 mg) |
| Niacinamide | 18 mg | 113% (16 mg) |
| Pantothenic acid | 6 mg | 100% (6 mg) |
| Pyridoxine (Vitamin B6) | 1.9 mg | 136% (1.4 mg) |
| Biotin | 30 µg | 60% (50 µg) |
| Folic acid | 600 µg | 300% (200 µg) |
| Cyanocobalamin (Vitamin B12) | 2.6 µg | 100% (2.5 µg) |
| Ascorbic acid | 60 mg | 75% (80mg) |
| Copper | 1.15 mg | 100% (1 mg) |
| Iodine | 250 µg | 167% (150 µg) |
| Iron | 27 mg | 193% (14 mg) |
| Selenium | 50 µg | 100% (55 µg) |
| Zinc | 10 mg | 100% (10 mg) |

**Family-level**

1. **Conditional cash-transfer:** A monthly cash transfer of ZAR 280.00 (equivalent to ~£12) will be transferred directly into a bank card issued to the primary caregiver of the adolescent participant co-resident in the same household. The transfer will be monthly, conditional on both caregiver and adolescent engaging with the CHW during their monthly home-based visit. The cash is intended to modestly supplement the child support grant and other government social grants, so enabling poor, food insecure households to make more costly and diverse food choices. ‘Nudging’ household-level behaviour change may have positive spill-over effects to other members of the household.
2. **Health literacy:** CHWs will provide caregivers with information and resources around food choices for better nutrition (e.g. the ‘Eatwell Guide’ to improve dietary diversity).
3. **Support:** CHW will support the primary caregiver to access social grants that they are also eligible to receive.

**Adolescents who become pregnant**: Those in the treatment arm continue with the intervention. In addition, CHWs will support young women to link early to and regularly attend antenatal services, support parental involvement during pregnancy, and continue the intervention as before the pregnancy. The MMN supplement regimen will continue throughout pregnancy unless the adolescents elect to rather take the antenatal clinic provided supplements. We will provide information for them to share with their antenatal clinic nurses so as to minimise risk of over supplementation.

**Control arm with non-health specific intervention:** To minimise biases such as special attention given to adolescents in the intervention arm and potentially greater attrition in the control arm, there will be repeated contacts with adolescents in the control arm through a call centre approach (infrastructure in place in both sites). Call centre personnel will deliver a monthly telephonic programme on *Life skills for your future* that will cover: (i) **Education for your future** (importance of education; guidance in selecting school subjects), (ii) **Civic rights and responsibilities** (how to apply for an identity document; what does it mean to vote?), (iii) **Financial education** (savings & budgets; how to set up a bank account; applying for an educational bursary), and (iv) **Navigating social media** (internet; various social media; benefits and harms of social media). Rapid pregnancy testing kits will be freely available for participants. For control participants who become pregnant during the course of the trial, call centre assistants will link them with usual-care antenatal services provided by local primary care clinic

## Study visits

| **Table 1. Data collection timeline** (adapted from protocol). | | | | | | | | |
| --- | --- | --- | --- | --- | --- | --- | --- | --- |
|  | | **Enrolment** | | | **Post-randomisation** | | | **Exit** |
| **TIMEPOINT** | | Screening | Baseline | Allocation | Month  0-18  (Max 24) | Pregnant  <28 week | Postnatal  <1 month | 18-24 Months |
| **ENROLMENT:** | |  |  |  |  |  |  |  |
| **Eligibility screen (**Height & weight to derive BMI) | | X |  |  |  |  |  |  |
| **Informed consent** | | X |  |  |  |  |  |  |
| **Randomisation** | |  |  | X |  |  |  |  |
| **TRIAL:** | |  |  |  |  |  |  |  |
| **Intervention Delivery Period** | |  |  |  | X |  |  |  |
| **Comparator Delivery Period** | |  |  |  | X |  |  |  |
| **ASSESSMENTS:** | |  |  |  |  |  |  |  |
| **NON-PREGNANT PARTICIPANTS** | |  |  |  |  |  |  |  |
| **Physical** | Height & weight (BMI); blood pressure; DXA-derived whole body composition (fat mass, fat free soft tissue mass; visceral adiposity) |  | X |  |  |  |  | X |
| **Biomarkers** | Hemoglobin (HemoCue); fasting glucose (Randox); insulin (Immulite); Q-Plex micronutrient array (haemoglobin, vitamin A, inflammatory markers, ferritin, soluble transferrin receptor) |  | X |  |  |  |  | X |
| **Nutrition** | Dietary practices; determinants of fruit and vegetable intake, nutritional knowledge, Eating Attitudes (EAT-26), household food security; vitamins B1, B2, B6, B12, C, D & E plus folate metabolites & lipid profile (multiplex panel) |  | X |  |  |  |  | X |
| **Lifestyle** | Household socio-demographic survey; substance use; objective accelerometry measures of physical behaviours: physical activity (min/day); sedentary behaviour (min/day); sleep (hrs/day) |  | X |  |  |  |  | X |
| **Health & wellbeing** | Self-regulation; health survey (infections, medical causes of underweight); mental health (PHQ9), anxiety (GAD-7), trauma (Adverse Child Events Scale), social support |  | X |  |  |  |  | X |
| **PRIMARY CAREGIVER** | |  |  |  |  |  |  |  |
| **Physical** | Height, weight (BMI); BP |  | X |  |  |  |  | X |
| **Health & wellbeing** | Parenting (Alabama parenting survey) |  | X |  |  |  |  | X |
| **PARTICIPANTS THAN BECOME PREGNANT** | |  |  |  |  |  |  |  |
| ***PREGNANCY*** | |  |  |  |  |  |  |  |
| **Physical** | Gestational weight gain, blood pressure |  |  |  |  | X |  |  |
| **Biomarkers** | Fasting glucose at 28 weeks |  |  |  |  | X |  |  |
| **Health & wellbeing** | Frequency of ANC visits, pregnancy complications (clinic data) |  |  |  |  | X |  |  |
| ***POSTNATAL*** | |  |  |  |  |  |  |  |
| **Physical** | Birth weight &length body composition (DXA) of infant |  |  |  |  |  | X |  |
| **Health & wellbeing** | Delivery complications Edinburgh postnatal depression scale |  |  |  |  |  | X |  |

## Outcomes

### Primary outcomes

Change in BMI standard deviation score (SDS) aligned to the target direction – i.e. increase in BMI for underweight, decrease in BMI for overweight – from baseline to follow-up at 18-24 months. As the trial is examining the efficacy of the intervention to address the triple burden of malnutrition (in underweight group increasing BMI; overweight group reducing BMI), the primary outcome variable is both appropriate and innovative.

### Secondary outcomes

We will examine the change in anaemia (iron status) as a secondary outcome. Blood pressure and blood glucose will also be key secondary outcomes and will be compared between arms. For those in the intervention group who become pregnant, maternal fasting glucose concentrations around 28 weeks and neonatal adiposity data at delivery (DXA-derived) will be compared with control pregnant participants and their offspring.

### Safety outcomes

- Any Serious Adverse Events during study period including:
  - Adolescent adverse events
  - Childhood adversity
  - Mortality

## Randomisation and blinding

## Randomisation: Participants are individually randomised 1:1 using a computer-generated sequence with variable block sizes to preserve allocation concealment. Randomisation, supported by the Cambridge Epidemiology and Trials Unit will be stratified by location (Agincourt/Soweto) and BMI status (underweight or overweight) and carried out using pre-calculated codes in sealed electronic envelopes displayed on a tablet to ensure allocation concealment. The participant herself will “select” between the two sealed options by actively tapping the envelope of choice.

## Blinding: Investigators and the research data-collection team are blinded to group assignment, which remains securely locked and inaccessible. The data management team may unblind a participant only at the request of the Data Monitoring Committee or the ethics committee for safety or mandated reporting. Blinding is maintained by (i) restricting database permissions, (ii) training staff and participants to avoid revealing allocation, and (iii) conducting intervention and control activities in separate areas.

## Sample size

For the **primary outcome**, with a total of 1248 adolescent girls (624 overweight: 312 intervention, 312 control; 624 underweight: 312 intervention, 312 control), there will be 90% power to detect a significant difference in arms of mean target-aligned change in BMI standard deviation score of 0.2 (gain in the underweight group, loss in the overweight/obese group) at the 2-sided 5% level. This assumes a standard deviation of change in BMI SDS of 0.687 calculated using data from adolescent girls in Agincourt assessed at two time points (mean ages 11.5 and 13.6 years), and expected 20% attrition. This degree of change in BMI SDS is equivalent to approximately 2 kg weight loss for girls with overweight and 1 kg higher weight gain in girls with underweight, assuming average height (further details about the sample size calculations, including scenario analyses, in table), compared to controls.

For **secondary outcomes**, the sample also provides 90% power at the 2-sided 5% level to detect a 2.1 mmHg difference in systolic blood pressure between intervention and control arms at follow-up, based on standard deviation of 10 mmHg as reported in young adult black Soweto female adolescents.

Regarding the **impact of the intervention on pregnancy outcomes**, rates of pregnancy in Soweto and Agincourt are high within the age group we plan to study (45.7/1000-person years during the 4-year follow up period). From the sample of 1248 girls we therefore anticipate ~208 pregnancies, which provides 80% power to detect a difference of 24% of a standard deviation (SD) (Intervention vs. Control) in any parameter at the two-sided 5% level, and 90% power to detect a 27.5% difference. These differences are equivalent to ~0.1 mmol/L in maternal fasting glucose at 28 weeks, 0.9-1.0% in neonatal percent body fat, and 0.6-0.7 mm in neonatal sum of skinfolds.

# Statistical analysis

**Analysis Populations**

- **Intention-to-treat (ITT):** All randomised participants analysed according to assigned arm, irrespective of adherence.
- **Modified ITT (mITT):** ITT excluding participants with no post-baseline outcome data for the specific endpoint (used in sensitivity checks).
- **Per-protocol (PP):** ITT excluding major protocol deviations (e.g., ineligible at baseline; unblinded outcome assessment; predefined non-adherence threshold
- **Pregnancy analysis cohort:** Randomised participants with a *pregnancy detected during the trial* and with the relevant maternal/neonatal outcome; analyses remain by original randomisation to avoid post-randomisation selection bias

**Primary Outcome**

**Target-aligned change in BMI-SDS** from baseline to 18–24 months.

- **Construction:**
  - Compute BMI-SDS (age-/sex-standardised using reference [specify WHO 2007; keep consistent across time points]).
  - For **underweight** stratum: aligned change = *(follow-up BMI-SDS – baseline BMI-SDS)* (higher is better).
  - For **overweight** stratum: aligned change = *(follow-up BMI-SDS – baseline BMI-SDS)* so that positive values always indicate improvement.
  - Combine strata for the primary analysis; include baseline BMI-SDS and baseline BMI stratum as covariates and test for interaction.

**Timing window:** primary follow-up closest to 18 months (allow 18–24 months).

**Pregnancy at measurement:** If a participant is pregnant at the 18–24-month visit or ≤6 months postpartum, the BMI-SDS at that visit is considered **not comparable**; preferred approach uses the last pre-pregnancy BMI-SDS within the window. If unavailable, treat as missing. Sensitivity: include all observed values regardless of pregnancy status.

**Secondary Outcomes**

- **Anaemia / iron status:** haemoglobin (g/L), ferritin (µg/L), soluble transferrin receptor, transferrin saturation; anaemia defined per WHO thresholds appropriate for age/altitude/smoking; iron deficiency per biomarker criteria with inflammation adjustment if CRP/AGP available.
- **Blood pressure:** Systolic and diastolic BP (mmHg); mean of last two of three seated measures using validated device.
- **Blood glucose:** Oral Glucose Tolerance Test or fasting plasma glucose (mmol/L) at follow-up.
- **Pregnancy subgroup endpoints:**
  - Maternal fasting glucose at ~28 weeks (±2 weeks).
  - Neonatal adiposity: percent body fat (DXA-derived) and sum of skinfolds (mm).

**Safety Outcomes**

- **SAEs** (ICHE2A/ICH-GCP definitions), **psychosocial harms** (adolescent adverse events, reported childhood adversity during follow-up where triggered), and **mortality** (all-cause).

**Estimands Framework (Primary)**

- **Population:** Randomised adolescent girls meeting inclusion criteria.
- **Treatment:** Assigned intervention vs control.
- **Variable:** Target-aligned change in BMI-SDS at 18–24 months.
- **Intercurrent events:** Pregnancy, protocol deviations, loss to follow-up.
- **Strategy:**
  - **Pregnancy:** *Treatment-policy* estimand for ITT (effect regardless of pregnancy), with missing-data handling as per Section 10 and sensitivity analyses with pre-pregnancy values.
  - **Non-adherence:** Treatment-policy for primary; PP as sensitivity.
  - **Missing data:** Multiple imputation under MAR; δ-adjusted MNAR sensitivity.
- **Summary measure:** Mean difference (intervention – control) on aligned BMI-SDS scale.
- **Population-level contrast:** ANCOVA / linear model adjusted for randomisation stratifiers and baseline BMI-SDS.

**General Analysis Principles**

- Two-sided α=0.05 for the primary outcome.
- Secondary outcomes: effect sizes and 95% CIs will be reported
- Continuous outcomes summarised as mean (SD) or median (IQR); binary as n/N (%) and risk difference, risk ratio, and odds ratio where relevant.
- All models will include **site** and **baseline BMI stratum** (stratification factors) and **baseline value of the outcome** when available.
- Imputation of baseline socio-demographic covariates if required to retain cases in MI.

**Statistical Methods**

**Primary Analysis**

**Model:** Linear regression (ANCOVA) of aligned BMI-SDS change on randomised arm, adjusting for baseline BMI-SDS, site, BMI stratum, and age at baseline.
Change_aligned ~ Arm + Baseline_BMI_SDS + Site + BMI_Stratum + Age

- **Primary contrast:** adjusted mean difference with 95% CI and p-value.
- **Heterogeneity by stratum:** include *Arm × BMI_Stratum* interaction (pre-specified). If p<0.10, present stratum-specific effects; otherwise, present pooled.
- **Sensitivity (repeated-measures):** Linear mixed-effects with random intercept for participant and fixed effects for time (baseline, 12m if available, 18–24m), Arm, Arm×Time; includes all valid time points (handles unequally spaced data).

**Secondary Analyses**

- **Anaemia/iron biomarkers (continuous):** ANCOVA as above; log-transform skewed biomarkers (e.g., ferritin), report geometric mean ratio.
- **Anaemia (binary):** Log-binomial regression (or Poisson with robust variance if non-convergence), adjusted for randomisation stratifiers and baseline Hb.
- **Systolic/diastolic BP:** ANCOVA adjusted for baseline BP, and randomisation stratifiers
- **Fasting glucose (non-pregnant cohort):** ANCOVA adjusted for baseline fasting glucose (if measured) or HOMA surrogates; otherwise baseline BMI-SDS and age.

**Pregnancy Analyses (Post-Randomisation Subgroup)**

Pregnancy is an intercurrent event potentially affected by treatment. To limit selection bias:

- **Primary pregnancy analyses:** ITT on the *pregnancy cohort*, adjusting for baseline predictors of pregnancy as covariates
- **Models:**
  - Maternal fasting glucose at 28w: weighted ANCOVA (adjust for baseline age, BMI-SDS, site, socio-economic index, prior pregnancy, and any baseline glycaemia markers).
  - Neonatal adiposity (percent fat, skinfolds): weighted ANCOVA; additionally adjust for gestational age at scan/delivery and infant sex.
- **Sensitivity:** ANCOVA without the additional adjustments (except for the randomisation stratifiers)..

**Subgroup Analyses (pre-specified; interaction tested at α=0.10)**

- **Site:** Agincourt vs Soweto.
- **Baseline BMI-SDS (continuous) & category:** underweight vs overweight (already above).
- **Age bands:** 13–15 vs 16–19 (or study-specific).
- **Socio-economic tertiles.**

For each, fit an interaction term (Arm × Subgroup) in the primary model; present estimates and 95% CI by level if interaction suggests heterogeneity.

**Per-Protocol and Adherence Analyses**

- **PP definition:** no critical deviations (eligibility, prohibited co-interventions), and **adherence ≥ pre-defined threshold** (e.g., ≥70% of scheduled sessions/contacts completed or composite adherence score ≥ pre-set).
- **Analysis:** same model as ITT, restricted to PP set.
- **Dose–response:** exploratory continuous adherence variable in the ITT using instrumental variable (2SLS) with randomisation as instrument (assumptions stated), plus complier average causal effect (CACE) if assumptions plausible.

**Interim Analyses, Data Monitoring, and Stopping**

- **Interim efficacy:** None planned.
- **Safety monitoring:** DSMB reviews unblinded aggregate safety at pre-specified intervals.
- **Stopping rules:** For safety concerns only, at DSMB discretion guided by clinical judgment; no formal alpha-spending.

**Protocol Deviations**

Pre-define major vs minor deviations. Major include: randomisation errors; baseline ineligibility; unblinded outcome assessment; use of prohibited interventions; missing primary outcome outside allowed window; pregnancy handling inconsistent with Section 5.1. Deviations logged before database lock; PP set defined accordingly.

**Descriptive & Baseline Tables**

Levels of missing data will be reported with reasons where available; baseline characteristics will be summarised in participants with and without missing values of the primary outcome

**Data Management & Quality**

- **Data capture:** REDCap/eCRF with edit checks; double range checks for primary/secondary outcomes.
- **Outliers:** Pre-specified plausibility bounds (e.g., SBP 70–190 mmHg; fasting glucose 2–20 mmol/L). Values outside trigger query; if confirmed, retained; if implausible, set missing.
- **Blind review:** T-flaw and data consistency checks conducted under masked arm labels before code freeze.

**Safety Analysis**

- **Definitions:** AE/SAE per ICH-GCP. Relatedness and expectedness judged by blinded clinical investigator where possible.
- **Summaries:** Number (%) with ≥1 AE/SAE; event counts and incidence rates (per 100 person-years if relevant); by system organ class and preferred term.
- **Comparisons:** Risk ratio (or rate ratio) with 95% CI; Fisher’s exact for rare events.
- **Psychosocial harms:** Pre-specified items (e.g., distress triggers), summarised similarly; urgent escalation pathway described in the protocol.

**Sensitivity & Supplementary Analyses**

- **Alternative primary metric:** Analyse raw BMI change (kg/m²) and BMI-SDS without alignment but with Arm×BMI_stratum interaction to demonstrate concordance.
- **Trimmed means:** 10% trimmed-mean difference for robustness to heavy tails.
- **Instrumental variable / CACE:** Explore effect among compliers (Section 9.5).
- **Contamination:** If control uptake of similar services is measurable, include a contamination covariate; perform bias-analysis scenario.

**Reporting**

- CONSORT flow diagram.
- Primary and secondary results reported as described
- All deviations from the SAP flagged in the final report.

**Reproducibility**

- **Code (STATA):** Version-controlled (Git) analysis scripts; seeded random number generation for MI/bootstraps; scripts and outputs archived.
- **Data lock & sign-off:** Analytic dataset Locked v1.0; SAP signed by PI, lead statistician, and sponsor before unblinding.
